# When is good, good enough? Methodological pragmatism for sustainable guideline development

**DOI:** 10.1186/s13012-015-0222-4

**Published:** 2015-03-06

**Authors:** George P Browman, Mark R Somerfield, Gary H Lyman, Melissa C Brouwers

**Affiliations:** The BC Cancer Agency, Vancouver Island Centre, Victoria BC and the School of Population and Public Health, University of British Columbia, Vancouver, BC Canada; American Society of Clinical Oncology, 2318 Mill Road, Suite 800, 22314 Alexandria, VA USA; Hutchinson Institute for Cancer Outcomes Research, Fred Hutchinson Cancer Research Center, Seattle, WA USA; University of Washington, Seattle, WA USA; McMaster University and the Escarpment Cancer Research Institute, Hamilton, ON Canada

**Keywords:** Evidence-based practice, Guideline development, Methodology, Framework

## Abstract

**Background:**

Continuous escalation in methodological and procedural rigor for evidence-based processes in guideline development is associated with increasing costs and production delays that threaten sustainability. While health research methodologists are appropriately responsible for promoting increasing rigor in guideline development, guideline sponsors are responsible for funding such processes.

**Discussion:**

This paper acknowledges that other stakeholders in addition to methodologists should be more involved in negotiating trade-offs between methodological procedures and efficiency in guideline production to produce guidelines that are ‘good enough’ to be trustworthy and affordable under specific circumstances. The argument for reasonable methodological compromise to meet practical circumstances is consistent with current implicit methodological practice. This paper proposes a conceptual tool as a framework to be used by different stakeholders in negotiating, and explicitly reporting, reasonable compromises for trustworthy as well as cost-worthy guidelines. The framework helps fill a transparency gap in how methodological choices in guideline development are made. The principle, ‘when good is good enough’ can serve as a basis for this approach.

**Summary:**

The conceptual tool ‘Efficiency-Validity Methodological Continuum’ acknowledges trade-offs between validity and efficiency in evidence-based guideline development and allows for negotiation, guided by methodologists, of reasonable methodological compromises among stakeholders. Collaboration among guideline stakeholders in the development process is necessary if evidence-based guideline development is to be sustainable.

“Dans ses écrits, un sàge Italien/Dit que le mieux est l’ennemi du bien.”

(In his writings, a wise Italian says that the best is the enemy of the good.)

Voltaire (Francois-Marie Arouet)

## Background

Evidence-based clinical practice guidelines (guidelines) that use the most rigorous methods can help inform clinical and policy decisions [1]. In the scientific enterprise, there is an imperative that we ensure new research builds appropriately on existing studies and that the current state of the science is well described to ethically justify the effort and expense of new studies. Indeed, within the guideline field, considerable effort has been directed towards ensuring that guidelines are of high quality [2], trustworthy [3], and implementable [4]. These goals typically translate to increasing demands on the scientific and procedural rigor employed in guideline development, and as a result, the threshold for acceptable methodological standards continues to be raised [2-7].

The evidence-based health care movement has harnessed and honed the methods of the evaluation and decision sciences to enhance the rigor with which we generate, critically evaluate, interpret, and apply clinical scientific knowledge for health care practice and policy decisions. This has been extended in the guideline context to consider the population perspective, guideline applicability, editorial independence, and the like. There are many tools and methods to support optimal guideline development, for example, the Appraisal of Guidelines for Research & Evaluation (AGREE) II [2], the Guideline International Network (GIN) Standards [6], the Institute of Medicine (IOM) Standards [3], and Guidelines 2.0 [7]. These methods play an influential and, from many perspectives, a welcome role in bringing greater accountability and credibility to practice recommendations. Such rigor is important to minimize biases that can creep into each step in the generation, reporting, and interpretation of evidence considered to formulate valid recommendations that can be implemented. But perhaps it is time to ask if the incremental price of applying escalating methodological standards in all circumstances has exceeded the incremental benefits. This paper argues for reasonable methodological compromise to meet practical circumstances consistent with current implicit methodological practice.

## Discussion

### Increasing demands in clinical practice guideline development

Increased methodological expectations translate into increased guideline development time, costs, and delays in their release. For example, the AGREE II, a commonly used quality rating instrument that also informs reporting and development, has 23 quality criteria [2]. The IOM Standards, a tool commissioned by the United States Congress to articulate the methods for the development of guidelines and recommendations, is composed of eight core standards underpinning 21 components [3]. Finally, the Guidelines 2.0 guideline development checklist comprises 18 themes and 146 steps [7]. Direct costs for guideline development can be high, as much as $200,000 per guideline in the United States [3,5]. Are these costs for a good-quality, trustworthy, implementable guideline worth the added benefits under all circumstances? Past a certain point, as yet undefined, to what extent do these additional methodological expectations lead to more trustworthy guidelines, better policy and practice, and better health outcomes? Could failure to meet expectations lead to greater downstream costs in the implementation of recommendations that may be more prone to bias, thus negating cost savings achieved from a more efficient methodological approach?

These are critical and pragmatic questions for guideline developers who create the documents, for those who use guidelines and must wait for recommendations, for those who fund guideline development and need to ration limited resources, and for those ultimately responsible for the implementation of recommendations. The short answer to these questions is that we simply do not know at what point increasing methodological rigor leads to appreciably more valid and implementable recommendations that, in turn, lead to better outcomes or more affordable care.

The evidence underpinning the value of available methodological tools or quality features as a means to reduce bias varies considerably [8]. For example, the inadequacy of blinding or allocation concealment is positively correlated with larger magnitudes of effect [9]. However, the strength of the relationship between the inclusion of a methodological tactic and the capacity to mitigate bias varies or is unknown. Furthermore, there is no common evaluation framework upon which new methodological tools are tested and no agreed upon outcomes that should be considered in determining if a new procedural step is worth the added time, effort, and potential expense. For example, while increasing the perceptions of guideline trustworthiness by clinicians is an appropriate tactic for buy-in as a prerequisite to promoting appropriate practice change, is it a sufficient measure upon which to justify the time and expense of a bit of extra rigor in guideline development procedures, or should there be a more direct link between procedural rigor and measures such as appropriate practice change and clinical outcome improvement?

### The interface between guideline methodology and stakeholder concerns

Methodologists have been the main drivers of continuously increasing procedural rigor in guideline development. However, the burden of escalating demands for rigor has been borne by others whose perspectives need to be taken into account to ensure timely completion of recommendations, continued guideline funding, and ongoing participation by guideline panel members (often voluntary). We contend that it is time for the guideline community, as a whole, to ask whether more efficient and affordable processes for guideline development can be used with credible results, towards achieving a better balance between rigor and pragmatism that addresses the needs of all stakeholders who are affected by guidelines. To this end, and consistent with the spirit of transparency and accountability that are at the core of an evidence-based guideline strategy, we argue for greater collaboration among methodologists and other guideline developers, researchers, consumers, funders, and other stakeholders in the application of guideline development methods. Arguably, there should be less emphasis on blind adherence to common, putative standards whose generalizability and impact are uncertain.

**Figure 1:**
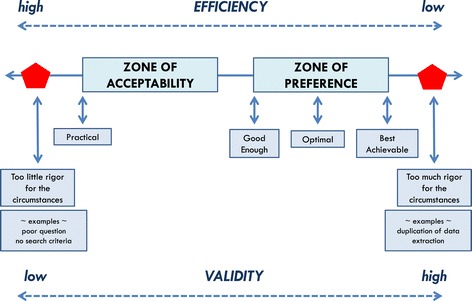
**The negotiation tool.** The Efficiency-Validity Methodological Continuum for sustainable guideline development.

Respecting the multi-stakeholder interests in guidelines would require negotiations among stakeholders, guided by methodologists, about acceptable methodological standards that are sensitive to the circumstances for which a guideline is being developed. To facilitate negotiation among stakeholders about methodological trade-offs in guideline development and their potential risks to validity, we offer a conceptual tool, the *Efficiency-Validity Methodological Continuum* (Figure 1). The goal of the tool is not to set new standards but to provide direction on how explicit and transparent negotiations can occur regarding the differential application of existing standards, and how choices may translate into variation in resource demands, validity, and implementation. The principle of ‘when good is good enough’ is used to support this direction.

### When is a guideline ‘good enough’?

The ‘good enough’ principle is a useful paradigm for beginning to address the balance between rigor and pragmatism in guideline development, while also taking into account (a) the inherent level of uncertainty about the evidence base and impacts associated with methodological expectations in guideline development or (b) where evidence is certain, the risk threshold for tolerating compromise in validity to gain efficiency. The principle of ‘good enough’ reflects the notions that continuous improvement will happen, that we must cope with a messy world that defies perfection, that one starts and chooses processes or materials whose ‘bugs’ are already known, and that judgment is required when defining what is good enough [10,11].

For those concerned with the erosion of quality associated with this approach, it has been observed that ‘Good enough has nothing to do with mediocrity; it has to do with rational choices, as opposed to compulsive behavior’ [11]. Moreover, the ‘good enough’ principle is not a radical change from what currently happens in the methodological culture, as seen with the plethora of critical appraisal instruments [2,12,13]. For example, it is common within systematic reviews to use less-than-ideal methods such as accepting language of publication limitations or accepting literature-based, as opposed to individual patient data meta-analyses, despite our knowledge of validity trade-offs [13-15]. Risks to validity when making compromises can be handled by shifting towards a more explicit discourse that involves sufficient consultation with all stakeholders and to fill the transparency gaps in methodological choices that already exist implicitly.

### The Efficiency-Validity Methodological Continuum: a negotiating tool for methodological trade-offs for more efficient guideline development

The circumstances that pose difficulties for highly rigorous guideline development are variable enough that it should be up to the relevant stakeholders to determine what constitutes an acceptable trade-off between rigor and efficiency in either developing or using a particular guideline. Figure 1 illustrates the model upon which the tool can be built. The guideline development approach used should be explicit about the methodological choices made by articulating the possible risks to validity and the ability to use recommendations, tactics to preserve credibility, and the gains in efficiency. As can be seen, the extreme right and left areas of the continuum in the figure are zones to avoid. The one extreme would arise if the methodological choices are suboptimal or an unconvincing rationale is provided for why some methodological steps are skipped: while potentially efficient, there is a consensus among most stakeholders that validity is compromised too much. For example, the failure to provide an explicit and researchable guideline question or the failure to articulate eligibility criteria to direct the study selection may fall into this situation. The other extreme would occur in circumstances where overemphasis on methodological issues would require additional expense and demand procedures where the evidence base to support them is lacking, or where there are little data to show impact on important outcomes. For example, extracting data in duplicate or searching the reference lists of identified studies may fall into this category.

Zones of ‘preference’ and ‘acceptability’ along the continuum (Figure 1) both reflect the ideal to be achieved in developing a valid evidence-based guideline contrasted to an approach to achieve efficiency in terms of time, effort, and costs with reasonable methodological compromise. The continuum implies that methods employed to maximize efficiency through methodological shortcuts may come with risks to validity. Conversely, striving for maximum validity may come at an unreasonable price in terms of expense and delay, thus compromising implementability. Most guideline developers will want to be somewhere between these two extremes, as close to the right (validity) as reasonably practical, with acceptable compromises to validity and implementability to be negotiated among stakeholders. The key when choosing an approach is transparency in terms of what compromises are agreed to, the costs, and how this is reported.

The proposed ‘efficiency-validity trade-off continuum’ is intended to raise awareness within organizations and among guideline sponsors and developers about the prospect that, as stakeholders, they have choices to negotiate among themselves towards credible compromises without delegating all methodological choices to the methodologist. Where there is little research-derived information that meets minimal standards for health care evidence, but where there is a demand or a need for guidance, consensus approaches may be most appropriate. The framework here may be modified for that purpose, negotiating an optimal balance between informal and formal consensus methods [16].

### How might this framework be used in practice and what is its potential impact?

We envision the ‘efficiency-validity trade-off continuum’ as an opportunity for promoting collaborative negotiation within multi-representative guideline development panels at the stage of protocol development for an evidence-based guideline to determine what is acceptable for a given project. Questions from the good enough quality paradigm [11] may serve as useful questions to facilitate the discussion (Table 1). In terms of what is ‘acceptable,’ we need to be aware that trade-offs are made on behalf of patients, the key stakeholders in the guideline enterprise who, therefore, should be represented on the guideline development panel where the negotiation for trade-offs is taking place. These circumstances may include the funding available, time considerations, the urgency of the need for guidance, and the intended use of the guideline (to advise clinical practice, influence system-level policy, incorporation into decision support systems, etc.). The threshold or limits of the boundaries for the zones of acceptability or preference along the continuum are determined by the negotiating partners and cannot be objectively prescribed for everyone nor generalized to all circumstances. Moreover, one may imagine that as methodological innovations and advancements are made that make the development process more efficient and less expensive, this too would shift thresholds and decisions about acceptability.

**Table 1:** **Potential factors and perspectives that could be used in stakeholder negotiations**

**Factors**	**Perspectives**
Benefits of specific guideline methodologies	Stakeholders (e.g., patients, developers, end-users)
Problems of specific guideline methodologies	Purpose of guideline
Quality, credibility, and implementability of resulting guideline document	Available time frame
	Alternatives and their consequences
Logistics of changing the methodologies (e.g., adding or eliminating steps)	Overall guideline quality, credibility, implementability

Adapted from Bach [11].

We postulate that using such a negotiating tool will over time limit unnecessary costs and, therefore, encourage increased investment in guidelines; opportunities towards further methodological refinement; and improved consensus about quality, trustworthiness, implementability, and ‘cost-worthiness’ of evidence-based guidelines. We predict furthermore that failure to address all stakeholder concerns around appropriate levels of investment in guidelines that are circumstance-sensitive will gradually erode the evidence-based guideline movement. This erosion is already demonstrated through the rising popularity and credibility of alternative, more affordable, and largely consensus-based models and by reasonable concerns that the efforts and costs associated with rigorous evidence-based methods may often produce recommendations that are not substantially different from those resulting from less rigorous and less costly approaches [17-19].

## Summary

We propose a re-examination of the trend towards ever-increasing methodological standards for guideline development with respect to the costs, efforts, and delays involved for the benefits gained. We suggest that decisions about guideline development procedures be circumstance-sensitive and therefore negotiated among all stakeholders who may be affected, rather than left solely to the research methodologist. We offer a conceptual tool, the ‘Efficiency-Validity Methodological Continuum,’ to help guideline stakeholders (sponsors/payers, clinicians, methodologists, and patient representatives) to respectfully negotiate credible compromises in methodological rigor imposed by practical constraints in developing a guideline. Compromises are already being made implicitly through the use of quality rating scales for individual studies and existing systematic reviews and guidelines, few of which are without flaws. Based on negotiated explicit and transparent decisions, guideline consumers can judge whether a guideline has achieved a level of quality, trustworthiness, and implementability that is ‘good enough.’
